# Supplementary material for: Unveiling the Bioactive Potential of the Invasive Jellyfish Phyllorhiza punctata Through Integrative Transcriptomic and Proteomic Analyses
Source: Biomolecules. 2025 Aug 4;15(8):1121. doi: 10.3390/biom15081121 (PMC12383608; doi:10.3390/biom15081121)
Supplement: Supplementary file 1 [file biomolecules-15-01121-s001.zip › Suplementary Material/FileS3/PEPFOLD4/XP_065058797_701/clusters.html]

 PEP-FOLD clusters


Download archive of all models  here   

|  |  |  |
| --- | --- | --- |
| # | file | sOPEP |
| # cluster 1 - Size: 1 |  |  |
|  | XP\_065058797\_701-model1 | -15.502 |
| # cluster 2 - Size: 4 |  |  |
|  | XP\_065058797\_701-model2 | -13.4892 |
|  | XP\_065058797\_701-model2.1 | -13.0017 |
|  | XP\_065058797\_701-model2.2 | -12.8845 |
|  | XP\_065058797\_701-model2.3 | -12.6605 |
| # cluster 3 - Size: 91 |  |  |
|  | XP\_065058797\_701-model3 | -13.0274 |
|  | XP\_065058797\_701-model3.1 | -13.0181 |
|  | XP\_065058797\_701-model3.2 | -13.0064 |
|  | XP\_065058797\_701-model3.3 | -12.9802 |
|  | XP\_065058797\_701-model3.4 | -12.9642 |
|  | XP\_065058797\_701-model3.5 | -12.9536 |
|  | XP\_065058797\_701-model3.6 | -12.9109 |
|  | XP\_065058797\_701-model3.7 | -12.9075 |
|  | XP\_065058797\_701-model3.8 | -12.9075 |
|  | XP\_065058797\_701-model3.9 | -12.9075 |
|  | XP\_065058797\_701-model3.10 | -12.9075 |
|  | XP\_065058797\_701-model3.11 | -12.9075 |
|  | XP\_065058797\_701-model3.12 | -12.9051 |
|  | XP\_065058797\_701-model3.13 | -12.9051 |
|  | XP\_065058797\_701-model3.14 | -12.9001 |
|  | XP\_065058797\_701-model3.15 | -12.8971 |
|  | XP\_065058797\_701-model3.16 | -12.8633 |
|  | XP\_065058797\_701-model3.17 | -12.8616 |
|  | XP\_065058797\_701-model3.18 | -12.8578 |
|  | XP\_065058797\_701-model3.19 | -12.8578 |
|  | XP\_065058797\_701-model3.20 | -12.8562 |
|  | XP\_065058797\_701-model3.21 | -12.8558 |
|  | XP\_065058797\_701-model3.22 | -12.8558 |
|  | XP\_065058797\_701-model3.23 | -12.8508 |
|  | XP\_065058797\_701-model3.24 | -12.8322 |
|  | XP\_065058797\_701-model3.25 | -12.8311 |
|  | XP\_065058797\_701-model3.26 | -12.8311 |
|  | XP\_065058797\_701-model3.27 | -12.8311 |
|  | XP\_065058797\_701-model3.28 | -12.8231 |
|  | XP\_065058797\_701-model3.29 | -12.8132 |
|  | XP\_065058797\_701-model3.30 | -12.8049 |
|  | XP\_065058797\_701-model3.31 | -12.7884 |
|  | XP\_065058797\_701-model3.32 | -12.7831 |
|  | XP\_065058797\_701-model3.33 | -12.7797 |
|  | XP\_065058797\_701-model3.34 | -12.7627 |
|  | XP\_065058797\_701-model3.35 | -12.755 |
|  | XP\_065058797\_701-model3.36 | -12.7466 |
|  | XP\_065058797\_701-model3.37 | -12.7304 |
|  | XP\_065058797\_701-model3.38 | -12.7195 |
|  | XP\_065058797\_701-model3.39 | -12.7112 |
|  | XP\_065058797\_701-model3.40 | -12.7112 |
|  | XP\_065058797\_701-model3.41 | -12.7026 |
|  | XP\_065058797\_701-model3.42 | -12.7024 |
|  | XP\_065058797\_701-model3.43 | -12.6826 |
|  | XP\_065058797\_701-model3.44 | -12.6825 |
|  | XP\_065058797\_701-model3.45 | -12.6794 |
|  | XP\_065058797\_701-model3.46 | -12.6615 |
|  | XP\_065058797\_701-model3.47 | -12.6611 |
|  | XP\_065058797\_701-model3.48 | -12.6608 |
|  | XP\_065058797\_701-model3.49 | -12.66 |
|  | XP\_065058797\_701-model3.50 | -12.6485 |
|  | XP\_065058797\_701-model3.51 | -12.6482 |
|  | XP\_065058797\_701-model3.52 | -12.6345 |
|  | XP\_065058797\_701-model3.53 | -12.6314 |
|  | XP\_065058797\_701-model3.54 | -12.6275 |
|  | XP\_065058797\_701-model3.55 | -12.623 |
|  | XP\_065058797\_701-model3.56 | -12.6187 |
|  | XP\_065058797\_701-model3.57 | -12.6098 |
|  | XP\_065058797\_701-model3.58 | -12.6067 |
|  | XP\_065058797\_701-model3.59 | -12.6046 |
|  | XP\_065058797\_701-model3.60 | -12.6037 |
|  | XP\_065058797\_701-model3.61 | -12.5848 |
|  | XP\_065058797\_701-model3.62 | -12.5806 |
|  | XP\_065058797\_701-model3.63 | -12.5694 |
|  | XP\_065058797\_701-model3.64 | -12.5685 |
|  | XP\_065058797\_701-model3.65 | -12.5665 |
|  | XP\_065058797\_701-model3.66 | -12.5619 |
|  | XP\_065058797\_701-model3.67 | -12.5581 |
|  | XP\_065058797\_701-model3.68 | -12.5581 |
|  | XP\_065058797\_701-model3.69 | -12.5545 |
|  | XP\_065058797\_701-model3.70 | -12.5538 |
|  | XP\_065058797\_701-model3.71 | -12.5442 |
|  | XP\_065058797\_701-model3.72 | -12.54 |
|  | XP\_065058797\_701-model3.73 | -12.533 |
|  | XP\_065058797\_701-model3.74 | -12.5102 |
|  | XP\_065058797\_701-model3.75 | -12.5073 |
|  | XP\_065058797\_701-model3.76 | -12.5011 |
|  | XP\_065058797\_701-model3.77 | -12.5009 |
|  | XP\_065058797\_701-model3.78 | -12.4948 |
|  | XP\_065058797\_701-model3.79 | -12.4917 |
|  | XP\_065058797\_701-model3.80 | -12.4665 |
|  | XP\_065058797\_701-model3.81 | -12.439 |
|  | XP\_065058797\_701-model3.82 | -12.433 |
|  | XP\_065058797\_701-model3.83 | -12.4132 |
|  | XP\_065058797\_701-model3.84 | -12.379 |
|  | XP\_065058797\_701-model3.85 | -12.3587 |
|  | XP\_065058797\_701-model3.86 | -12.3529 |
|  | XP\_065058797\_701-model3.87 | -12.3244 |
|  | XP\_065058797\_701-model3.88 | -12.2444 |
|  | XP\_065058797\_701-model3.89 | -11.9402 |
|  | XP\_065058797\_701-model3.90 | -11.8734 |
| # cluster 4 - Size: 4 |  |  |
|  | XP\_065058797\_701-model4 | -12.7165 |
|  | XP\_065058797\_701-model4.1 | -12.6952 |
|  | XP\_065058797\_701-model4.2 | -12.6952 |
|  | XP\_065058797\_701-model4.3 | -12.6013 |
|  |  |  |
